# Exploring the pathogenesis of age-related macular degeneration: A review of the interplay between retinal pigment epithelium dysfunction and the innate immune system

**DOI:** 10.3389/fnins.2022.1009599

**Published:** 2022-11-03

**Authors:** Josephine H. C. Wong, Jessica Y. W. Ma, Andrew I. Jobling, Alice Brandli, Ursula Greferath, Erica L. Fletcher, Kirstan A. Vessey

**Affiliations:** Department of Anatomy and Physiology, The University of Melbourne, Melbourne, VIC, Australia

**Keywords:** age related macular degeneration (AMD), retinal pigment epithelium (RPE), mononuclear phagocyte (MP), microglia, dendritic cell, macrophage, para-inflammation, inflammation

## Abstract

Age-related macular degeneration (AMD) is a leading cause of irreversible vision loss in the older population. Classical hallmarks of early and intermediate AMD are accumulation of drusen, a waste deposit formed under the retina, and pigmentary abnormalities in the retinal pigment epithelium (RPE). When the disease progresses into late AMD, vision is affected due to death of the RPE and the light-sensitive photoreceptors. The RPE is essential to the health of the retina as it forms the outer blood retinal barrier, which establishes ocular immune regulation, and provides support for the photoreceptors. Due to its unique anatomical position, the RPE can communicate with the retinal environment and the systemic immune environment. In AMD, RPE dysfunction and the accumulation of drusen drive the infiltration of retinal and systemic innate immune cells into the outer retina. While recruited endogenous or systemic mononuclear phagocytes (MPs) contribute to the removal of noxious debris, the accumulation of MPs can also result in chronic inflammation and contribute to AMD progression. In addition, direct communication and indirect molecular signaling between MPs and the RPE may promote RPE cell death, choroidal neovascularization and fibrotic scarring that occur in late AMD. In this review, we explore how the RPE and innate immune cells maintain retinal homeostasis, and detail how RPE dysfunction and aberrant immune cell recruitment contribute to AMD pathogenesis. Evidence from AMD patients will be discussed in conjunction with data from preclinical models, to shed light on future therapeutic targets for the treatment of AMD.

## Introduction

**FIGURE 1:**
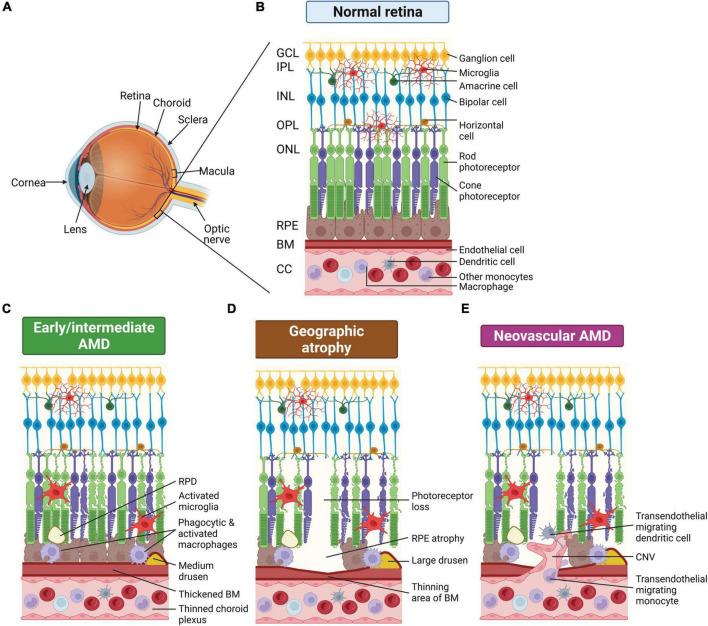
Schematic of changes that occur in the eye of age-related macular degeneration (AMD) patients. **(A)** Schematic render of the eye showing posterior ocular tissues affected in AMD, expanded in subsequent panels. **(B)** Healthy posterior eye, showing transverse section of the retinal layers, the retinal pigment epithelium (RPE), Bruch’s membrane (BM), and vascular supply (the choriocapillaris, CC). The location of the mononuclear phagocytes (MPs) is indicated including retinal tissue resident microglia associated with the neuronal synaptic layers (nerve fiber layer MPs not shown) and MPs in the choroid, including dendritic cells, macrophages and other monocytes. **(C)** Schematic of early/intermediate AMD showing thickening of BM, deposition of extracellular waste around the RPE (drusen and reticular pseudodrusen, RPD), thinned choroid, and changes in MPs, including activation of microglia within the retina. **(D)** Schematic of late-stage AMD, geographic atrophy, showing loss of photoreceptors and RPE cells, thinning of BM in regions of cell loss, and deposition of large drusen. Microglia within the retina are activated and activated macrophages on the choroidal side are associated with the damaged basal RPE/BM. **(E)** Schematic of late-stage neovascular AMD showing choroidal neovascularization (CNV), loss of photoreceptors and RPE cells, thinning of BM in regions of cell loss and deposition of large drusen. Microglia within the retina are activated and peripheral circulating monocytes and dendritic cells are activated and associated with damaged RPE and are also able to enter the retina via new leaky vessels. This illustration was started from a BioRender Template and content and stylistic modifications were made.

Age-related macular degeneration (AMD) is a leading cause of irreversible blindness in people over 50 years of age. Patients initially present with distortions in fine visual perception, and as the disease develops, vision symptoms progress to loss of central vision impacting abilities such as reading, face recognition and driving (Hassell et al., 2006; Rattner and Nathans, 2006). In severe cases, vision loss can progress to complete legal blindness impacting an affected individual’s independence and quality of life (Hassell et al., 2006; Rattner and Nathans, 2006). AMD is characterized by chronic and progressive degeneration of the light sensitive neurons of the retina, the photoreceptors, and their support cells, the retinal pigment epithelium (RPE) (Miller, 2013). In the early and intermediate stages, AMD is diagnosed based on the presence and size of extracellular deposits, called drusen, which accumulate between the RPE and choroidal blood supply, and also by the presence of pigmentary abnormalities in the RPE. Abnormal waste deposits within RPE cells, called lipofuscin, and extracellular deposits between the photoreceptor and the RPE, called reticular pseudodrusen (RPD) are also manifestations of the disease (Holz et al., 2001; Greferath et al., 2016). Late AMD is generally classified into two groups: dry AMD or geographic atrophy (GA), and wet or neovascular AMD (nAMD) (Figure 1). Around 80–90% of AMD cases including intermediate and late-stage GA are the dry, atrophic form, which is generally a slowly progressing disease, where over time the atrophic areas become larger and confluent (Ferris et al., 1984). nAMD occurs when abnormal choroidal vessels grow under the RPE or when retinal blood vessels sprout within the retina. Vision loss in nAMD is accelerated as these abnormal vessels leak and bleed into the macula, leading to sudden vision loss due to macular edema and direct damage to the photoreceptors, and over time this leads to fibrous scarring (Ciulla et al., 2001; Lim et al., 2012; Little et al., 2018). At present, there are no effective treatments to slow progression from early and intermediate AMD to late stages of the disease (GA and nAMD). While there are no treatments for GA, nAMD is treated with anti-vascular endothelial growth factor (VEGF) drugs (Solomon et al., 2019) and while short-term visual acuity improvement is often observed, vision loss over the longer term occurs despite treatment in most cases (Rofagha et al., 2013). Effective treatments for slowing progression of early AMD to the late stages are urgently required.

In AMD, RPE and photoreceptor dysfunction occur alongside accumulation of waste materials in the subretinal space (RPD) and between the RPE and choroid (drusen), initiating recruitment of resident and systemic immune cells. The dominant immune cell type observed is mononuclear phagocytes (MPs) and these are rarely seen in the outer retina and RPE-Bruch’s membrane complex in age-matched, non-diseased eyes, which implicates MPs in the pathology of AMD. MPs play a primary role in phagocytosis of pathological material in AMD. Resident MPs in the retina are called microglia and are postulated to remove cell debris in the subretinal space to protect photoreceptors and the RPE against injury and death (Langmann, 2007; Ambati et al., 2013). Additionally, drusen deposition between the RPE and choroid and failure of the RPE can cause breakdown of the blood retinal barrier, which can lead to recruitment and infiltration of systemic circulating MPs, the myeloid cells in the peripheral blood circulation comprising monocytes, macrophages and dendritic cells (Ransohoff and Cardona, 2010; Luhmann and Ali, 2012). In the early stages, AMD presents as a disease of low-grade chronic inflammation, called para-inflammation, which is denoted by the perpetual recruitment and non-resolving presence of MPs (Xu et al., 2009; Leveillard et al., 2019). Indeed, RPE dysfunction and non-resolving inflammation remain two prominent hypotheses for the pathogenesis of AMD (Xu et al., 2009; Guillonneau et al., 2017). In this review, we explore how the RPE and innate immune cells maintain retinal homeostasis, and consider the role of RPE dysfunction and aberrant immune cell recruitment in AMD pathogenesis.

## Clinical definitions and treatment of age-related macular degeneration

### Early and intermediate age-related macular degeneration

Diagnosis of AMD is made based on clinical fundus examination or assessment of color fundus photographs usually in conjunction with imaging for pigmentary changes and changes in the structure of the retina. Several classification systems for AMD are available. The Beckman classification suggests small drusen with a size of ≤63 μm are considered as a sign of normal aging and are associated with a low risk of progressing to AMD, with around 1% of population having small drusen that may progress to late AMD in 10 years (Ferris et al., 2013). Medium drusen between >63 and ≤125 μm are considered as an early sign of AMD and suggest an increased risk of progressing to large drusen and late AMD (Ferris et al., 2013). Other phenotypes that are associated with early to intermediate AMD include RPE pigmentary changes and the formation of RPD deposits in the subretinal space. There are no treatments for early or intermediate AMD to markedly slow or completely arrest development to late stages of the disease.

### Geographic atrophy

Patients with early or intermediate AMD may develop either of the two advanced forms of the disease: GA or nAMD. In GA there is a sharply demarcated area of RPE hypo-pigmentation with a diameter of at least 175 μm and visible choroidal vessels on fundus examination (Bird et al., 1995). GA usually originates in a region around the perimeter of the fovea and expands across other parts of the parafovea (Sarks et al., 1988; Sunness, 1999; Wolf-Schnurrbusch et al., 2008). The hypo-pigmentary abnormalities of the RPE correlate with RPE atrophy and photoreceptor deterioration, involving the disorganization and loss of photoreceptor inner and outer segments and eventually photoreceptor death (Young, 1987; Sarks et al., 1988; Kim et al., 2002). Additionally, age-dependent accumulations of autofluorescent lipofuscin, which are hyperfluorescent waste deposits within the RPE, is another characteristic of AMD (Hogan, 1972; Sarks, 1976; Young, 1987). Diagnosis of GA can be aided by examination of fundus autofluorescence (FAF). Regions of hyper-autofluorescence correlate with increased deposition of autofluorescent lipofuscin, subretinal autofluorescent material including RPD, and changes in the photoreceptor photopigment. Large regions of hypo-autofluorescence correlate with decreased lipofuscin due to loss of RPE cells, generally indicating regions of late-stage AMD. There are no approved therapies for atrophic AMD.

### Neovascular age-related macular degeneration

The hallmark of nAMD is the pathological proliferation of new blood vessels into the macula, with subsequent macular edema, subretinal hemorrhage and end-stage fibrous scarring. Based on the origin of the abnormal vessel growth, nAMD can be classified into three subtypes: types I and II choroidal neovascularization (CNV), and type III which occurs in the retina, retinal angiomatous proliferation (RAP) (Sarks et al., 1997; Yannuzzi et al., 2008). CNV is the aberrant extension of vessels from the choroid passing into either the sub-RPE space (occult CNV; type I), or further anteriorly into the subretinal space following loss of the RPE (classic CNV; type II) (Sarks et al., 1997). RAP refers to the growth of blood vessels originating from the inner retinal circulation, which continues into the subretinal space, and merges with the choroidal circulation to form an anastomosis between the retinal and the choroidal vessels (Yannuzzi et al., 2001, 2008). Regardless of the nAMD subtypes, the new blood vessels can be leaky, causing serous fluid and blood to accumulate in the subretinal space and the neural retina, often leading to rapid and severe vision impairment and potential detachment of the retina. Furthermore, fibrotic changes occur in late stages of nAMD and this is denoted by a well-circumscribed fibrotic white or yellow scar on fundus examination (Stevens et al., 1997; Rogers et al., 2002; Bloch et al., 2013). Fibrosis may cause dysfunction and degeneration of the photoreceptors, through hindering vascular supply and provoking RPE degeneration (Bloch et al., 2013).

Therapeutic approaches currently approved by the United States Food and Drug Administration and the Australian Therapeutic Goods Administration for arresting nAMD are intravitreal injections of drugs that block VEGF, a growth factor which induces proliferation of vascular endothelial cells and angiogenesis (Solomon et al., 2019). Whilst this treatment enables short-term visual improvement in the majority of treated patients, one-third of nAMD patients, especially those with classic CNV, do not show any arrest of vision loss or improvement in visual acuity after receiving anti-VEGF therapy (Bloch et al., 2013; Rofagha et al., 2013). Moreover, patients with RAP frequently develop GA following anti-VEGF treatment (McBain et al., 2011). These poor visual outcomes are associated with subfoveal fibrosis development, which remains untreatable (Rofagha et al., 2013).

## The role of the retinal pigment epithelium in retinal homeostasis and retinal pigment epithelium changes in aging and age-related macular degeneration

### Retinal pigment epithelium cellular morphology

**FIGURE 2:**
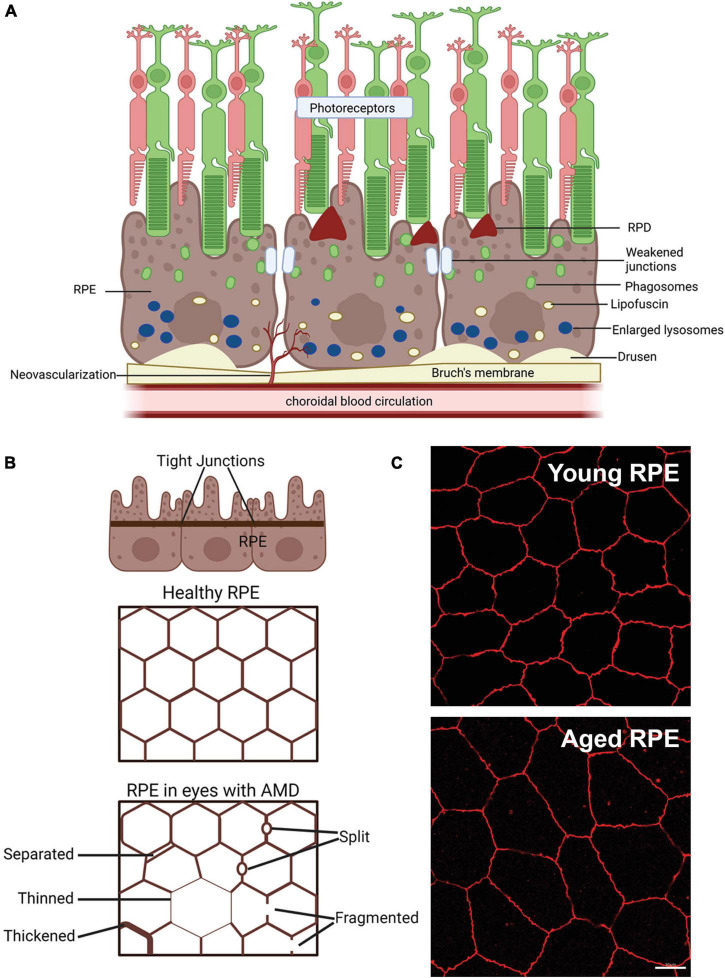
Schematic of changes that occur in the retinal pigment epithelium (RPE) of age-related macular degeneration (AMD) patients. **(A)** Schematic of changes within the RPE in late-stage AMD, including weakening of tight junctions that form the blood retinal barrier, deposition of lipofuscin and enlarged failed lysosomes. Deposition of extracellular waste around the RPE, large drusen on the basal side of the RPE are apparent and reticular pseudodrusen (RPD) on the apical side may also manifest. **(B)** Schematic showing changes in the tight junctions of the RPE in AMD in flat mount view. Healthy RPE are hexagonal in shape and tight junctions form a continuous connection around each RPE cell. In AMD, RPE cells may be larger due to cell loss and tight junctions may be separated, thinned, thickened, split or fragmented, modified from Ach et al. (2015). **(C)** Example of the effects of aging on the mouse RPE. Tight junction ZO-1 labeling of young (3 month) and old (22 month) mouse RPE, shows increased RPE cell size in the aged tissue, suggesting cell loss with age. Some of this illustration was started from a BioRender Template and content and stylistic modifications were made.

Dysfunction and degeneration of the RPE is one of the key features of AMD. In the following sections the function of the RPE in maintaining retinal homeostasis and how it changes in normal aging vs. in AMD will be considered (Figure 2). The RPE is a continuous monolayer of terminally differentiated epithelial cells that lies between the neural retina and choroidal blood supply (Boulton and Dayhaw-Barker, 2001; Strauss, 2005 for general reviews of RPE structure and function). The RPE apical surface has long and thin microvilli that interface with the photoreceptor outer segments. The basal surface of the RPE has numerous infoldings and the basolateral membrane forms a part of Bruch’s membrane, comprising the inner wall of the choroid. The RPE nucleus is located basally and a number of unique organelles are present inside the cells to support function (Boulton and Dayhaw-Barker, 2001; Strauss, 2005). These organelles include mitochondria, phagosomes and lysosomes which are associated with renewal of the photoreceptor outer segments and are found at the basal side of the cell. Additionally, the RPE contains melanosomes, which are located either at the base of or within the apical process of the RPE depending on diurnal phase, and these are important in absorbing stray light to improve visual acuity (Boulton and Dayhaw-Barker, 2001; Strauss, 2005). The size and phenotype of the RPE cells vary with retinal eccentricity in human eyes. The cells at the macula are smaller, with a diameter varying from 7 to 11 μm. They become larger and irregular away from the macula, reaching 60 μm or more in the peripheral retina (Salzmann and Brown, 1912; Ach et al., 2014).

When eyes age normally, there is a decrease in the density of RPE cells representing cell loss (Gao and Hollyfield, 1992; Bhatia et al., 2016). In addition, some RPE cells become multinucleated with age particularly in the periphery, representing either cell fusion or failed attempts at cell division (Ts’o and Friedman, 1967). Changes in the RPE cytoskeleton are also associated with aging. RPE cells lose their hexagonal shape and become larger, elongated and more irregular with aging (Tarau et al., 2019). This is particularly evident in the macula and far peripheral retina (Rashid et al., 2016), while in the far periphery, RPE cells become thinner and more irregular with age (Bhatia et al., 2016; Rashid et al., 2016). Despite the change in geometry and decreased cell density, the intact monolayer is maintained during normal aging.

In eyes with AMD, in addition to regions of RPE cell loss, additional RPE structural changes occur that are different from those seen in normal aging. Prior to cell loss, the RPE cells in the context of AMD lesions show morphological changes, becoming concave and round (Ach et al., 2015). Uneven thinning, thickening, separation, and splitting are observed along with fragmentation of the cytoskeletal component, F-actin (Tarau et al., 2019). The separation and fragmentation of F-actin suggests RPE-regulated outer blood-retinal barrier (discussed later) may be weakened in AMD.

### Bruch’s membrane

As mentioned above, the RPE contributes to Bruch’s membrane, a layered semi-permeable extracellular matrix tissue delineated by and including the choriocapillaris and RPE basement membranes (Hogan and Alvarado, 1967; Sarks, 1976; Kliffen et al., 1997; Curcio and Millican, 1999). Bruch’s membrane consists of five layers: the basement membrane of the RPE, the inner collagenous zone, a central band of elastic fibers, the outer collagenous zone and finally the basement membrane of the choriocapillaris (Nakaizumi et al., 1964). Nutrients and oxygen from the choroid and waste from the RPE must be transported across Bruch’s membrane to maintain retinal homeostasis. It also acts as a support for RPE physical adhesion and restricts RPE and choroidal vessel migration (Bhutto and Lutty, 2012).

During normal aging, Bruch’s membrane thickens. In the early stages of AMD, Bruch’s membrane thickness increases above that seen in normal aging. Further analysis of Bruch’s membrane using electron microscopy shows that abnormal deposits appear above and below the RPE basement membrane, called basal laminar and basal linear deposits respectively, in early/intermediate AMD (Hogan and Alvarado, 1967; Sarks, 1976; Kliffen et al., 1997; Curcio and Millican, 1999). The appearance of basal linear deposits correlates with large drusen observed in early/intermediate AMD and may constitute an early form of drusen (Curcio and Millican, 1999). This thickening process has been suggested to reduce nutrient and waste exchange between the RPE and the choroid, contributing to RPE and photoreceptor dysfunction in AMD. As AMD progresses to the late stages of the disease, Bruch’s membrane thins in areas where the choroid, RPE and photoreceptors are lost (McLeod et al., 2009).

### The blood retinal barrier

One of the primary functions of the RPE is providing nutrients and oxygen from the choroidal blood supply to the neural retina and in exchange, removing and recycling waste products from the retina, some of which are returned to the choroid (O’Leary and Campbell, 2021). To ensure controlled movement of molecules between the retina and fenestrated choroid, the RPE layer forms the blood retinal barrier in the outer retina, while endothelial cells, glia and pericytes contribute to the blood retinal barrier in the inner retina (O’Leary and Campbell, 2021). Tight junctions between neighboring RPE cells provide this dynamic barrier that controls the movement of crucial nutrients and waste between the neural retina and the choroid (Marmorstein, 2001; Rizzolo, 2014). The tight junctions predominantly include claudin family proteins, MARVEL family transmembrane proteins and junctional adhesion molecules (O’Leary and Campbell, 2021). These tight junctions prevent the infiltration of large molecules, pathogens and active immune cells from the peripheral blood circulation into the neural retina. The presence of Bruch’s membrane and an intact outer blood retinal barrier together with the anti-inflammatory and immunosuppressive factors released by the RPE cells, maintain the immune privilege of the outer retina (Sugita, 2009).

During healthy aging, tight junctions between the RPE remain intact, maintaining a continuous barrier between the choroid and the neural retina, however, the choroidal plexus does thin with age (Wakatsuki et al., 2015). In intermediate AMD, toxic plasma components, that should be excluded from the retina and are absent in healthy aged tissue become apparent, suggesting the blood retinal barrier is compromised prior to late-stage disease (Schultz et al., 2019). Specifically, plasma proteins: albumin, fibrinogen, immunoglobulin G (IgG), and complement component 9 (C9) are present in the retina of intermediate AMD patients (Schultz et al., 2019). In GA, the choroid plexus becomes thinner than observed in normal aging (Adhi et al., 2014) and even when there is no overt sign of exudative change, the blood retinal barrier is compromised with plasma proteins apparent in the retina (Schultz et al., 2019). A compromised blood retinal barrier is also observed in nAMD, evidenced by the pathological proliferation of leaky, new blood vessels. A recent study suggests altered granzyme B expression, which is produced by the RPE and choroidal mast cells, contributes to the breakdown of RPE tight junctions and the extracellular matrix in nAMD (Matsubara et al., 2020). Indeed, deterioration of RPE tight junctions and the blood retinal barrier is required for neovascular changes, as animal studies indicate upregulation of expression of angiogenic factors such as VEGF or angiopoietin 2 are not enough to induce new vessel growth and that loss of tight junctions is required (Oshima et al., 2004). Together these results suggest that deterioration of the RPE and the blood retinal barrier is an important pathological occurrence in intermediate and late-stage AMD, which is required for the formation of new blood vessels in nAMD.

### Retinal pigment epithelium immune regulation

The RPE contributes to immune regulation of the retina in several ways: generation of the outer blood retinal barrier, production of an immunosuppressive and anti-inflammatory microenvironment, and ability to sense and respond to pathogens. The RPE expresses a range of factors and receptors that play a role in protecting the retina from pathogens that may be present in the circulatory system. The RPE expresses pattern recognition receptors such as toll-like receptors (TLR), which sense molecular patterns associated with microbe pathogens (e.g., bacteria, viruses, and fungi) that may be present in the circulatory system (Kumar et al., 2004). To date 10 mammalian TLRs have been identified with 9 identified in human RPE cells (TLR 1-7 and 9 and 10) (Kumar et al., 2004). In addition to the TLRs, the RPE also expresses other pattern recognition receptors (e.g., Nucleotide-binding oligomerization domain, Leucine rich Repeat and Pyrin domain containing proteins; NLRP), complement components, and a range of cytokines, chemokines and growth factors which limit pathological insult (Detrick and Hooks, 2020). In response to pathogens and the presence of systemic immune cells, the RPE expresses antigen presenting receptors (e.g., major histocompatibility complex class I and II molecules; MHC 1 and II) (Liversidge et al., 1988; Osusky et al., 1997; Detrick and Hooks, 2020). However, the healthy RPE favors an immunosuppressive state. For example, RPE cells induce Fas ligand expression when in contact with either activated T-cells or MPs such as monocytes that induces apoptosis of these inflammatory cells (Jorgensen et al., 1998; Hettich et al., 2014). It is likely that the RPE produces a unique balance between an anti-inflammatory and pro-inflammatory environment to maintain optimal retinal function.

With age, changes in the immune response of the RPE occur, however, few studies have been completed on human samples. In RPE culture, aged cells show significantly increased production of cytokines, interferon (IFN)-γ, tumor necrosis factor (TNF)-α, interleukin (IL)-1α, IL-1β, IL-6, IL-8, IL-10 (Sreekumar et al., 2022) suggesting with age there is a shift toward to a more inflammatory environment. In AMD, there have been reported changes in RPE production of cytokines and chemokines that would likely alter the immunosuppressive environment of the outer retina. In AMD, transcriptome analysis of RNA sequencing (RNA-seq) data from human RPE of healthy control and AMD samples indicates changes in expression in a range of immune factors occur in late-stage disease (Saddala et al., 2019). Upregulation in TLR receptors, and their receptor cascades, complement receptors and a range of cytokines and chemokines and their receptors were identified in RPE from AMD patients (Saddala et al., 2019). Chemokines and their receptors have key roles in the movement of immune cells and altered expression of these factors are implicated in a wide range of inflammatory diseases including AMD. Chemokine ligands such as C-C motif chemokine ligand (CCL) 2 (CCL2), CCL3, CCL4, CCL13, CCL19, CCL21, chemokine C-X-C motif ligand (CXCL) 9 (CXCL9), CXCL10, CXCL16 and receptors C-C motif chemokine receptor (CCR) 1 (CCR1), CCR5, and CXCR6 were all identified as changing in the RPE of AMD patients (Saddala et al., 2019). Additionally, the alternative and classical complement pathways complement component 3 (C3), complement factor B (CFB), complement factor H (CFH), complement factor I (CFI), complement C1q A chain (C1QA) have been identified by RNAscope to change in GA (Demirs et al., 2021). The upregulation of complement factors and chemokines and associated receptors in the RPE suggests the RPE is generating a pro-inflammatory environment in late-stage AMD. The change in RPE expression of these immunogenic components would contribute to the recruitment of MPs between the RPE and choroid and to the subretinal space in AMD and this interaction will be considered in more detail below under the sections on MPs.

### Phagocytosis

Retinal pigment epithelium cells are one of the most phagocytic post-mitotic cells found in the body (Young, 1967, 1987). One RPE cell is in contact with 30–50 photoreceptors, that shed around 10% of their photoreceptor outer segment mass daily (Zinn and Benjamin-Henkind, 1979). The RPE carries out diurnal phagocytosis of these shed photoreceptor outer segments, and this process is required for maintaining optimal photoreceptor function (Strauss, 2005). Phagocytosis of the photoreceptor outer segment involves RPE binding, engulfment and phagocytosis, allowing recycling of vitamin A and fatty acids through the visual cycle and lipid cycling, respectively (Baehr et al., 2003). Following engulfment, the phagosomes bind with lysosomes, which contain enzymes allowing effective degradation of the photoreceptor outer segments.

The phagocytic ability of the RPE declines moderately with age, but it is greatly decreased in eyes with AMD when compared to age-matched normal RPE from human donors (Inana et al., 2018). Following binding and engulfment, RPE phagosomes of photoreceptor outer segments fuse with lysosomes to effectively recycle vitamin A derivatives and lipids. Failure in this process lead to lysosomal accumulations with age, which are greatly increased in AMD (Inana et al., 2018). These enlarged lysosomes contain undigested waste that accumulate as residual bodies, which are autofluroescent waste in the RPE, known as lipofuscin (discussed in more detail below in intracellular waste accumulations).

In animal models of AMD also, the RPE shows a similar decrease in phagocytic ability. This has been attributed in part to increased iron levels, as mRNA and protein expressions of several iron-regulatory molecules are significantly increased with age leading to iron accumulation. Excess iron is toxic to the RPE cells and impairs phagocytosis and lysosomal function (Chen et al., 2009). However, other processes, including changes in autophagy, have been implicated in slowing the phagocytosis process (Yu et al., 2018). The phagocytosis process and autophagy pathways share similar intracellular protein pathways and slowing of autophagy in the RPE with age may contribute to the slowing of processing of outer segment renewal by phagocytosis (Mitter et al., 2014; Yu et al., 2018).

### Autophagy

In addition to phagocytosis, the RPE carries out autophagy, a process by which cell components such as old mitochondria and waste products such as lipids and proteins are renewed by intracellular recycling (Kunchithapautham and Rohrer, 2007). Like the recycling of photoreceptor outer segments, this process also involves compartmentalizing intracellular waste within an autophagosome and binding with a lysosome to complete the degradation process. In general, autophagy becomes insufficient with age, either because autophagic flux is reduced or because there are too many cellular components that need to be removed from chronic cellular damage (Kroemer, 2015). Examination of control human donor specimens shows there is an age-related increase in autophagosome numbers and expression of autophagy proteins in the RPE (Mitter et al., 2014). RPE cells upregulate autophagy to enhance the renewal of different cellular components in response to a variety of insults with age. Yet, when the insult is prolonged and there are too many cellular components that need to be removed, autophagy becomes impaired. In line with this, autophagy proteins and autophagy flux are significantly reduced in RPE from human donors with AMD (Mitter et al., 2014; Golestaneh et al., 2017). Additionally, there is an accumulation of cytoplasmic debris, lipid and undigested protein aggregates tagged for autophagic degradation, confirming dysregulated autophagy in the RPE of eyes with AMD (Golestaneh et al., 2017).

Mice with impaired autophagy demonstrate age-dependent degeneration of the RPE with RPE pigmentation defects and RPE atrophy, hallmarks of late-stage AMD, observed. Furthermore, mice in which the autophagy pathway genes *ATG5* and 7 have been conditionally deleted from the RPE, show defects consistent with early AMD (Zhang et al., 2017), while other rodent models of AMD [apolipoprotein E4 (ApoE4) mice fed a high fat diet and apolipoprotein E (ApoE) null animals] show impaired autophagy in aged animals and RPE change consistent with early AMD (Mitter et al., 2014; Vessey et al., 2022). Failure in the autophagy pathways would lead to intracellular waste accumulation, which would contribute to RPE cell damage in AMD.

Impaired autophagy in the RPE has also been reported to promote inflammation. In RPE culture, mouse RPE cells with dysfunctional autophagy significantly increase secretion of inflammatory caspase-1, a marker for NLRP3 inflammasome activation, and cytokine IL-1β following co-culture with bone marrow derived macrophages (Liu et al., 2016). This indicates inflammasome activation in MPs follows defective autophagy in the RPE. Accumulation of macrophages with caspase-1 activation in the subretinal space was also detected after RPE and photoreceptor death in mice with impaired autophagy in the RPE (Liu et al., 2016). These data suggest that autophagy dysfunction in RPE cells can potentially trigger a series of inflammatory responses, including the influx of MPs and the activation of the inflammasome cascade, which may contribute to cell death in AMD.

### Lipid cycling

The photoreceptors require high levels of lipids, fatty acids and unesterified cholesterol and these are actively transported between the photoreceptors, RPE and choroidal blood supply (Tserentsoodol et al., 2006). For example, the RPE recycles phospholipid-esterified docosahexaenoic acid (DHA), which is otherwise primarily acquired from the diet, from shed rod outer segment disks to renew the function of the photoreceptors (Fu et al., 2021). In addition, fatty acids are used by photoreceptors to supplement their energy needs (Heckenlively et al., 2003; Joyal et al., 2016). Very low-density lipoprotein receptor (VLDLR) expressed by the RPE and photoreceptors, is critical for transport of lipids into the cell by anchoring ApoE triglyceride-rich lipoproteins. The fatty acid, palmitate, has been shown to be an energy source for photoreceptor mitochondria, likely as it yields three times the number of adenosine triphosphate (ATP) molecules than a single glucose molecule (Fu et al., 2021). To maintain the health of the retina, these lipids need to be recycled or transported from the RPE to the photoreceptors.

In AMD, abnormal lipid cycling has been suggested as a driver of AMD progression due to changes in lipid levels either through lifestyle factors or genetic mutations. In AMD eyes, accumulations of lipid droplets and lipid-derived components occur within the RPE cell (Curcio et al., 2009). Also, lipid pools develop as basal linear deposits, between the RPE basement membrane and the inner collagenous layer of Bruch’s membrane, occasionally form a “lipid wall” (Curcio et al., 2009). The presence of a “lipid wall” basal linear deposit would significantly reduce nutrient and oxygen transfer from the choroid to the RPE, impairing RPE function. The location and content of these basal linear deposits is suggested to represent soft drusen and highlights the importance of lipid cycling in the RPE in AMD.

Systemic changes in circulating lipids, genetic alterations in lipid processing and body mass have been found to have variable associations with risk of AMD development. A higher body mass index is an established risk factor for AMD development (Seddon et al., 2011). Interestingly evidences for systemic lipid changes as being associated with an increased risk of AMD are still not clear (Semba et al., 2019). A study of 177 serum lipids found no association to AMD status (Semba et al., 2019). It may even be those classic markers of “healthy” lipid levels in the serum, such as high density lipoprotein (HDL), low density lipoprotein (LDL) and low triglycerides may even incur increased risk of developing AMD (Grassmann et al., 2017). However, genetic association of increased risk of AMD with lipid transporters such as ApoE (Klaver et al., 1998) and changes in lipid processing within the RPE itself, may contribute to disease progression. Additionally, systemic changes in the carnitine shuttle, which is essential for mitochondrial β-oxidation of fatty acids, indicate that progressive dysfunction of mitochondrial fatty acid metabolism could be a key contributing factor to AMD progression (Mitchell et al., 2021).

### Intracellular waste accumulation: Lipofuscin and melanolipofuscin

Intracellular deposition of autofluorescent waste called lipofuscin occurs normally with age (Bianchi et al., 2013). It is believed to accumulate due to incomplete degradation of photoreceptor outer segments and slowing of autophagy resulting in accumulation of waste proteins and lipids contained within lysosomes and also failed lysosomes (residual bodies). Melanolipofuscin is lipofuscin that is associated with melanosomes present within the RPE and like lipofuscin, accumulates normally with age contributing to autofluorescence on FAF imaging (Ach et al., 2015). Excess accumulation of lipofuscin and melanolipofuscin occurs with age and in AMD and has been suggested to interfere with RPE function (Haralampus-Grynaviski et al., 2003; Rozanowska et al., 2004; Bianchi et al., 2013). In eyes with AMD, lipofuscin and melanolipofuscin redistribute within the RPE, sometimes forming large deposits or granulations within the cell, leaving other regions free from these accumulations (Ach et al., 2015). These deposits can result in cellular dysfunction as visible-light irradiation of lipofuscin causes lipid peroxidation, and production of hydrogen peroxide that can damage mitochondrial DNA in RPE cells (Rozanowska et al., 1995; Liang and Godley, 2003). Moreover, chronic oxidative stress induced by the photoreactive lipofuscin can contribute to age-dependent impairment of RPE phagocytosis (Olchawa et al., 2017). Intracellular accumulations of lipofuscin and melanolipofuscin in AMD would contribute to oxidative stress in the RPE, promoting RPE dysfunction (Haralampus-Grynaviski et al., 2003; Rozanowska et al., 2004).

### Extracellular waste accumulations: Drusen and reticular pseudodrusen

One of the important pathological manifestations of AMD is the presence of waste deposits in and around the RPE. Extracellular deposits in AMD include drusen, which accumulate between the RPE and the choroidal blood supply, and RPD, which are subretinal deposits that are observed in some cases of AMD. Histologically drusen are associated with RPE cellular changes and loss of RPE integrity (Schlanitz et al., 2019). Analysis of drusen composition has identified a variety of molecules, including RPE cellular components, immune- and/or inflammation-associated proteins, lipids and carbohydrates (Hageman et al., 2001). RPE derived components of drusen include: RPE fragments, lipofuscin and melanin, complement activator and amyloid beta (Aβ) (Sarks et al., 1999; Hageman et al., 2001; Johnson et al., 2002). Lipoprotein containing lipid transporter, ApoE is also proposed to be secreted by the RPE, and to be retained by the less permeable Bruch’s membrane, and may contribute to the formation of medium drusen (Mullins et al., 2000; Pikuleva and Curcio, 2014).

Immunogenic components are also apparent in drusen and may contribute to the recruitment of choroidal derived immune cells such as MPs. The presence of complement factors and several immune system proteins has been reported in drusen (Johnson et al., 2000; Mullins et al., 2000; Anderson et al., 2002). CFH and other complement components such as complement component 5 (C5) and C9 which are important for triggering innate immune cell activation and inhibition of self-damage have been identified in drusen (Mullins et al., 2000; Russell et al., 2000; Fett et al., 2012; Crabb, 2014). These findings correlate with genetic association studies, in which polymorphisms in the genes for complement components have been identified as a risk factor for AMD (e.g., CFH) (Edwards et al., 2005; Hageman et al., 2005), and hint that changes in MP interaction with complement-tagged cellular waste may be a driver of disease development. Additionally, vitronectin, a major drusen constituent, is a plasma and RPE derived extracellular matrix glycoprotein and may play a role in inhibiting complement-induced MP cell damage (Preissner, 1991; Hageman et al., 1999; Crabb et al., 2002). Cell membrane-associated protein complex human leukocyte antigen DR (HLA-DR) is also present in drusen and is thought to be derived from MPs, specifically dendritic cells from the choroid (Russell et al., 2000; Hageman et al., 2001). The presence of these immune factors likely contributes to the recruitment of choroidal derived MPs and accumulation of immune cells associated with drusen lesions in AMD (Combadière et al., 2007; Sennlaub et al., 2013; Levy et al., 2015; Silverman and Wong, 2018).

Unlike drusen, to date very little research has been completed on the make-up of RPD. Histologically, the subretinal presence of RPD is associated with RPE and photoreceptor disruption (Greferath et al., 2016). Clinical data from intermediate AMD patients indicate that RPD are associated with decreased photoreceptor function (Flamendorf et al., 2015), choroidal changes (Keenan et al., 2020), and over time with increased retinal thinning and degeneration (Chiang et al., 2020). The histological studies that have been undertaken show RPD to contain similar components to drusen such as photoreceptor outer segment debris including opsins; lipid in the form of unesterified cholesterol; RPE fragments (melanolipofuscin granules); ApoE, a protein important for lipid trafficking; vitronectin; and the immunogenic protein, CFH (Rudolf et al., 2008; Greferath et al., 2016; Wu et al., 2022). Interestingly, β-amyloid as well as a range of complement factors and immune markers, including C3, C5a, cluster of differentiation (CD) molecules CD11β and CD102, were not detected histologically and may not be present in RPD, highlighting a distinction between this type of deposit and classical drusen (Greferath et al., 2016). Further work is needed in this area to confirm the components of RPD and how they arise. However, despite the lack of certain immune related deposits, MPs particularly microglia/macrophages have found to be associated with RPD suggesting that the innate immune system is associated with the presence of these abnormal subretinal deposits (Greferath et al., 2016).

What is controversial about the origin of these extracellular deposits, drusen and RPD, is whether deposition is a causative event or consequence of RPE degeneration and MP recruitment. Early studies using electron microscopy suggest that primary degeneration of the RPE is partly accountable for the formation of drusen (Farkas et al., 1971; Green and Key, 1977). However, others have suggested that drusen hinder the diffusion of oxygen and nutrients from the choroid to the retina, driving RPE degeneration (Hageman et al., 2001; Anderson et al., 2002; Rattner and Nathans, 2006). The immunogenic composition of drusen and to some extent RPD, suggest they are extracellular waste that are tagged for removal by local MPs, but this process comes with a cost. The debris likely induces ongoing local inflammation and immune activation by gathering chemokines and cytokines with the potential for “collateral damage” to nearby healthy cells. It is likely that a combination of both RPE dysfunction teamed with MP failure to clear extracellular deposits with age that leads to continued accumulation of waste in the form of drusen and RPD, driving the cycle of disease progression. In the next sections, we will consider the role of MPs in healthy eyes and in AMD.

## Mononculear phagocytes and the ocular innate immune system

### Origins of ocular mononuclear phagocytes

Mononuclear phagocytes are a dominant cell population of the innate immune system and have been widely reported to participate in the homeostatic regulation and the pathogenesis of disease within the mammalian retina. They comprise four cell types from the myeloid lineage of blood cells: blood monocytes, dendritic cells, tissue macrophages and retinal microglia, all of which have been implicated in the pathogenesis of AMD (Ransohoff and Cardona, 2010). Blood monocytes are a population of circulating mononuclear leukocytes (white blood cells) originated from hematopoietic stem cells in the bone marrow (Geissmann et al., 2003; Ransohoff and Cardona, 2010). In the postnatal period, the stem cell-derived myeloid progenitors produced in the bone marrow give rise to common monocyte-dendritic cell progenitors. These progenitors are released into the peripheral blood circulation to form a reservoir of myeloid cell precursors, and are transported to tissues for cytokine-driven cellular differentiation (Geissmann et al., 2003; Ransohoff and Cardona, 2010). Dendritic cells share the same lineage as blood monocytes, also being derived from monocyte-dendritic cell progenitors, yet their differentiation from monocytes remains debatable (Geissmann et al., 2003; Ransohoff and Cardona, 2010). Macrophages can be derived from blood monocytes, or from undefined MP progenitors in the circulation. Microglia, despite being identified as a type of mononuclear leukocytes, and sharing many similar phenotypical and functional features as monocytes and macrophages, are derived from hematopoietic progenitors in the embryonic yolk sac during development (Mildner et al., 2007; Ginhoux et al., 2010). Reminiscent of brain microglia, retinal microglia originate from microglial precursors or circulating monocytes, which invade the retinal tissue via the vitreal surface or ciliary margin within the embryonic and early postnatal life before the blood retinal barrier is finalized (Barron, 1995; Marin-Teva et al., 1998; Santos et al., 2008; Ranawat and Masai, 2021).

### Change in peripheral blood monocytes in aging and age-related macular degeneration

**FIGURE 3:**
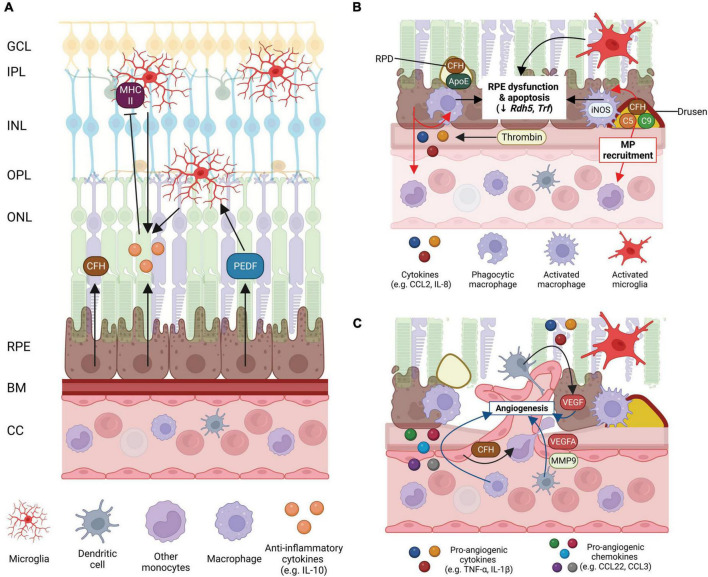
Schematic of mononuclear phagocyte (MP) interactions with the ocular tissues in health eyes and late-stage age-related macular degeneration (AMD). **(A)** Healthy posterior eye, showing transverse section of the retinal layers, the retinal pigment epithelium (RPE), Bruch’s membrane (BM), and vascular supply (the choriocapillaris, CC). The location of the mononuclear phagocytes (MPs) is indicated including retinal tissue resident microglia associated with the neuronal synaptic layers (nerve fiber layer MPs not shown) and MPs in the choroid, including dendritic cells, macrophages and other monocytes. RPE expression of immunosuppressive cytokines (e.g., IL-10), growth factors (e.g., PEDF) and immune factors (e.g., CFH) are shown. **(B)** Schematic of late-stage AMD, geographic atrophy, showing loss of photoreceptors and RPE cells, and deposition of large drusen. The RPE expresses chemokines, e.g., CCL-2 and IL-8 to recruit immune cells to areas of damage. Microglia within the retina are activated, recruited to damaged RPE cells and RPD and activated iNOS-positive macrophages on the choroidal side are associated with the damaged RPE and drusen containing immunogenic complement pathway factors. Activated MP interaction with the RPE can contribute to RPE dysfunction and apoptosis. **(C)** Schematic of late-stage neovascular AMD showing choroidal neovascularization (CNV), loss of photoreceptors and RPE cells, and deposition of large drusen. Microglia within the retina are activated. Peripheral circulating MPs, dendritic cells are activated and associated with damaged RPE releasing VEGF and MMP-9 to drive new vessel development and are also able to enter the retina via new leaky vessels. There is an increase in expression of pro-inflammatory chemokines and cytokines by MPs and the RPE which drives angiogenesis and contributes to RPE cell dysfunction and death. Some of this illustration was started from a BioRender Template and content and stylistic modifications were made.

Peripheral blood monocytes change with age, leading to an increased vulnerability to infection and the development of inflammatory diseases such as atherosclerosis and cancer and these changes may contribute to the development of AMD (Figure 3). Peripheral MP trafficking from bone marrow to blood, and responses to bacterial infection and phagocytosis are all reduced with age (Blacher et al., 2022). In intermediate and late-stage AMD, there is an increase in the number of peripheral blood monocytes suggesting these cells play a role in disease progression (Xue et al., 2021). In AMD, peripheral MP phagocytosis is significantly reduced irrespective of disease stage. Intermediate and late-stage AMD patients and those patients with RPD, all display reduced MP phagocytosis compared to healthy age-matched controls (Gu et al., 2021). Further investigation of expression of surface markers that are important in regulating leukocyte adhesion, migration, and phagocytosis (integrins CD11b and CD11c) show reduced expression in peripheral monocytes of late-stage AMD patients, both GA and nAMD (Gu et al., 2021). Importantly, these integrins form complexes with CD18 to form complement receptor 3 (CR3, CD11b/CD18) and complement receptor 4 (CR4, CD11c/CD18), respectively (Lukacsi et al., 2017). These receptors are both recognized to play an important role in phagocytosis and are reduced in peripheral MPs of late AMD. Further evidence supporting a reduction in peripheral MPs comes from work showing CD34, a marker of hemopoietic stem cells, is reduced in those with late AMD, implying that the capacity for differentiation of hemopoietic stem cells into monocytes is also reduced in those with late AMD (Gu et al., 2021). Alternatively, studies investigating the surface expression of molecules known to be important in phagocytosis, P2X purinoceptor 7 (P2X7) and CD33, report increased expression in monocytes of late-stage AMD patients (Gu et al., 2021). The role of P2X7 on MPs in AMD is further indicated by genetic studies which suggest inheritance of a loss of function P2X7 receptor variant combined with P2X purinoceptor 4 (P2X4) variant increases the risk of developing AMD, likely due to a reduction in the ability of peripheral blood monocytes and microglia to phagocytose debris (Gu et al., 2013). As MP phagocytosis of debris would be critical to removal of subretinal and drusen deposits, a reduction in this aspect of MP function would contribute to AMD development and progression.

As mentioned previously, chemokines and their receptors have key roles in the movement of immune cells and are implicated in a wide range of inflammatory diseases, including AMD. Evaluation of expression of four chemokine receptors (CCR1, CCR2, CCR5, and CXCR3) in CD14 + peripheral blood monocytes in patients with GA and nAMD showed that monocytes have increased expression of CCR5 in GA and that CXCR3 expression is increased in both GA and nAMD (Krogh Nielsen et al., 2020). CCR5 expression was low on monocytes of healthy controls as it is generally not expressed under homeostatic conditions (Krogh Nielsen et al., 2020), but an increase in expression on monocytes has been shown to occur during acute inflammation (Castanheira et al., 2019), suggesting a role for this chemokine receptor in ongoing inflammatory response in late-stage AMD. CXCR3 is involved in leukocyte migration and induces cytoskeletal and integrin changes that facilitate movement to areas of inflammation (Karin, 2020), suggesting that these cells may migrate differently in late-stage AMD. In line with this, in a smaller study (*n* = 9 AMD vs. *n* = 9 healthy controls) expression of other chemokine receptors involved in cell migration, CCR1 and CCR2 receptor expression on peripheral blood monocytes may be upregulated in late-stage nAMD (Anand et al., 2012), however this was not observed in a larger sample study (Krogh Nielsen et al., 2020). More work is required to carefully evaluate all the chemokine receptors expressed on peripheral blood MPs and determine if there are alterations in expression in AMD. Future work in this area may highlight important systemic changes in circulating MPs that contribute to AMD development and progression.

### Changes in retinal microglia in aging and age-related macular degeneration

The tissue resident MPs of the retina, microglia, are found in the nerve fiber layer, inner plexiform layer and outer plexiform layer of the healthy eye, where they are closely associated with the vasculature, neuronal synapses and glia. During aging, abnormal accumulation of microglia in the subretinal space occurs in both humans and mice (Xu et al., 2008; Ma et al., 2013). Microglia that accumulate in the subretinal space display a less ramified morphology with round cell bodies, shorter dendrites and less branching, suggestive of an activated phenotype (Xu et al., 2008; Damani et al., 2011; Ma et al., 2013). The recruitment of microglia to the subretinal space is thought to occur secondarily to RPE and photoreceptor dysfunction, to aid removal of photoreceptor outer segment debris (Combadière et al., 2007; Chinnery et al., 2012). Additionally, the activity of intraretinal microglia is altered with age. Time-lapse live imaging of resident microglia in the outer plexiform layer shows microglia have decreased process mobility in aged mice (Damani et al., 2011). Since aged microglia are less mobile, this may prolong the presence of microglia in the subretinal space, potentially contributing to photoreceptor and RPE pathology.

In AMD, the sustained presence of microglia/MPs in the subretinal space and microglial accumulation of lipofuscin may potentiate RPE injury and photoreceptor degeneration (Combadière et al., 2007; Xu et al., 2008; Chinnery et al., 2012; Ma et al., 2013). Like aged tissue, in AMD, subretinal microglia are bloated and have fewer processes suggesting activation (Figure 3; Penfold et al., 1997; Combadière et al., 2007). Histopathological examinations of human donors with GA show abnormal microglia/MPs in the subretinal space and the photoreceptor layer, which are enlarged, morphologically activated and contain photoreceptor components (rhodopsin) in their cytoplasm (Gupta et al., 2003; Combadière et al., 2007). Activated microglia/MPs may promote further photoreceptor degeneration in GA, as the loss of cones adjacent to atrophic regions has been linked to their accumulation in the subretinal space (Buschini et al., 2011; Eandi et al., 2016) and these activated microglia produce pro-inflammatory cytokines and nitric oxide which may be toxic to neurons (Gupta et al., 2003; Srinivasan et al., 2004; Zhang et al., 2005).

Evidence from mouse models where the blood retinal barrier is intact, but components of the immune system are mutated, hint at an important and specific role for subretinal microglia in age-related RPE and photoreceptor dysfunction. Mice lacking Ccl2, which is important for RPE recruitment of MPs (Ambati et al., 2003; Vessey et al., 2015); P2X7-receptor, which is important for MP phagocytosis (Vessey et al., 2017); and Cx3cr1 – mouse study not human, which is expressed solely by MPs (Combadière et al., 2007), all show subretinal microglia associated with RPE damage and retinal dysfunction that is reminiscent of early AMD in aged animals relative to age-matched controls. This suggests that perturbations in the function, recruitment and retention of subretinal microglia has the potential to contribute to RPE and photoreceptor failure with age. However, due to a lack of specific markers, differentiating subretinal microglia from infiltrating MPs is difficult. Despite evidence indicating a role for inflammation in AMD, the differential contribution of retinal microglia vs. peripheral monocytes/macrophages in the progression to the advanced stages of AMD in humans is unclear. Thus, in future sections, which detail the inflammatory changes in MPs in the subretinal space and their interaction with the RPE, microglia will be referred to as MPs unless their origin is explicitly clear.

### Maintenance of homeostasis via immune regulation

The healthy RPE secretes immunosuppressive factors that inhibit activation of MPs and even promotes macrophage death (Taylor et al., 2015). Peripheral blood MPs and resident choroidal MPs such as macrophages are present in the choroidal vasculature interface with Bruch’s membrane (Cherepanoff et al., 2010). However, the integrity of the blood retinal barrier blocks the entrance of peripheral blood MPs into the healthy retina. Within the retina, resident MPs, the microglia are found primarily in the synaptic layers of the retinal neurons and are not present in the photoreceptor layers or the sub-retinal space between the photoreceptor outer segments and the RPE. Hence, the subretinal space of healthy eyes is devoid of MPs, and there is no direct cell-cell contact between the MPs and RPE cells in the subretinal space.

The RPE contributes to the immune regulation of the retina via the release of anti-inflammatory factors including complement inhibitors (e.g., CFH) and cytokines, such as IL-10 (D’Orazio and Niederkorn, 1998; Langmann, 2007; Luo et al., 2013). IL-10 expression from MPs themselves has been implicated as playing an important role in maintaining homeostasis and immune privilege. For example, pigment epithelial derived factor (PEDF) released from the RPE increases IL-10 production by macrophages (Zamiri et al., 2006). Further recruitment and activation of MPs, particularly microglia is inhibited by IL-10, by reducing expression of antigen-presenting molecules such as monocyte MHC II (Figure 3A; D’Orazio and Niederkorn, 1998). Even under stress, the RPE tries to maintain an anti-inflammatory environment. Advanced glycation end-product (AGE) application, which induces oxidative stress, causes RPE cells in culture to upregulate expression of anti-inflammatory cytokines and receptors IL-9, IL-10, IL-13, and IL-1ra (Lin et al., 2013). IL-9 protects against RPE proliferation and apoptosis (Demoulin and Renauld, 1998) and IL-13 generally inhibits pro-inflammatory cytokine synthesis (Wynn, 2003). IL-1ra binds with comparable affinities for both IL-1α and IL-1β to the IL-1 receptor, without activating the receptor, playing a role in sopping up the overt inflammatory function of IL-1β (Gabay et al., 2010). The RPE cell also expresses Fas ligand which can induce apoptosis of inflammatory cells (Jorgensen et al., 1998). These immunomodulatory factors are likely a few of the mechanisms by which the RPE and MPs suppress MP recruitment and activation in the healthy retina.

### Mononuclear phagocyte migration to the retinal pigment epithelium and retention

In healthy eyes and with age, choroidal MPs such as macrophages are not found within Bruch’s membrane or closely associated with the RPE (Cherepanoff et al., 2010). However, in early AMD, MPs including macrophages are recruited to Bruch’s membrane drusen and basal laminar deposits and begin to express inducible nitric oxide synthase (iNOS), a marker of MP activation (Cherepanoff et al., 2010). As AMD progresses, in GA, MPs continue to express iNOS and to be associated with Bruch’s membrane. In early stages of subclinical CNV, MPs are associated with the new choroidal vessels (Cherepanoff et al., 2010). In late-stage CNV, MPs are found associated with Bruch’s membrane, the new vessels and within the retina. On the subretinal side, with age, MPs are occasionally found between the photoreceptor outer segments and RPE. However, in AMD, MPs are associated with RPD (Greferath et al., 2016; Wu et al., 2022) and the degenerating RPE (Wu et al., 2022). The proximity of MPs with the RPE waste deposits on both the choroidal and subretinal side in early and late AMD indicates the importance of these macrophages in waste clearance.

Damaged RPE cells and the deposition of waste material containing inflammatory molecules can induce the migration of MPs. RPE cells express surface receptors for sensing and responding to the exposure of cytokines and in turn rapidly release soluble chemoattractant inflammatory mediators to direct the trafficking of immune cells to the injured site (Elner et al., 1991; Hageman et al., 2001; Ma et al., 2017; Natoli et al., 2017). CCL2, also known as monocyte chemoattractant protein-1, is well known to be involved in the recruitment and retention of CCR2 bearing MPs to sites of dysfunction. CCL2 is present in higher concentration in the aqueous humor of human eyes with AMD (Kramer et al., 2012; Lechner et al., 2017). In cell culture studies, direct cell-cell contact between human RPE and inactivated monocytes causes a significantly larger production of CCL2 than cell co-culture with physical separation (Yoshida et al., 2001; Bian et al., 2003). This suggests that the presence of MPs associated with the RPE, drives further recruitment of MPs via the CCL2-CCR2 axis. Thrombin, a clotting factor which extravasates at sites of blood retinal barrier breakdown, can also enhance the secretion of CCL2 and IL-8 from RPE cells (Yoshida et al., 2001). Thus, the breakdown of the blood retinal barrier as occurs even in early AMD may predispose eyes to excessive recruitment of monocytes to sites of RPE dysfunction.

In cell culture studies, direct contact between human RPE cells and inactivated monocytes also causes the secretion of IL-8 by the RPE, compared to the co-culture of human RPE cells with monocytes separated by a filter (Yoshida et al., 2001; Bian et al., 2003). IL-8 is a cytokine associated with chemotaxis of immune cells to sites of damage and subsequent phagocytosis of damage, as well as being a mediator of angiogenesis (Koch et al., 1992; Harada et al., 1994). Polymorphisms in the IL-8 promotor, IL-8-251AA, are associated with an increased risk of AMD (Goverdhan et al., 2008). The upregulated secretion of IL-8 during a cell-cell contact co-culture suggests that like CCL2, RPE release of IL-8 is involved in recruiting MPs to sites of RPE dysfunction.

### Para-inflammation interactions between the retinal pigment epithelium and mononuclear phagocytes in aging and age-related macular degeneration

Once MPs are recruited to the RPE and retained, a process of para-inflammation ensues. Para-inflammation is an adaptive response in which tissue-resident or recruited MPs generate low-grade inflammation and is essential for maintaining tissue homeostasis and restoring tissue function. This process occurs with age and in early AMD, when the RPE and retina are stressed from a low-degree of noxious insults (Medzhitov, 2008; Xu et al., 2009). Since para-inflammation is thought to be an intermediate state between basal homeostasis and classic inflammation, upregulation of expression of anti-inflammatory molecules (discussed in the section on homeostasis) may help maintain retinal health by suppressing the inflammatory phenotypes of MPs (discussed in the section on pathological interactions below). In the early stages of AMD, there is likely to be a continuum of homeostatic and pro-inflammatory pathways activated.

Cell culture studies of MPs, co-cultured with RPE cells provide clues as to how these cells interact. Due to the immunosuppressive property of RPE cells, activated immune cells are likely to be eliminated when they are in close contact with RPE cells (Jorgensen et al., 1998). MPs have also been suggested to promote RPE cell survival and even proliferation, however, whether RPE cells are able to proliferate to repair areas of degeneration is not clear. It is generally accepted that RPE are terminally differentiated cells, but in mice, there is some evidence of proliferation in areas of lesions generated by a laser (Jobling et al., 2015) and some studies suggest that low grade proliferation in aged tissues may repair tissue damage, as a response to RPE cell death with age (Chen et al., 2016). Cell culture studies suggest that cell-cell contact between the RPE and monocytes/retinal microglia can induce the proliferation of RPE cells (Osusky and Ryan, 1996; Ma et al., 2009). However, this may prompt the RPE cells to lose integrity in morphology and intercellular adhesion, and form irregular clumps instead of a monolayer organization (Ma et al., 2009). Whether such an interaction whereby MPs have the potential to promote RPE survival and proliferation occurs *in vivo* is yet to be determined.

### Pathological interactions between the retinal pigment epithelium and mononuclear phagocytes in aging and age-related macular degeneration

The balance between para-inflammation and inflammation may be key to development of AMD. Genetic studies indicate regulation of the innate immune system’s alternative complement pathway confers increased risk of disease development. Indeed, an over-reactive alternative complement pathway due to mutations in CFH and C3, as well as downstream effectors such as C5 have been suggested to tip a patient into a “complement hyperinflammatory phenotype” (Whitcup et al., 2013). It is likely that failure to suppress the inflammatory MP response during basal para-inflammation contributes to disease progression in some cases of AMD. Over time, overt inflammatory interactions between MPs and the RPE occur and may drive AMD progression.

The direct interaction between RPE cells and MPs can induce pro-inflammatory cytokine production. Components of drusen from donor AMD patients and changes in accumulation of AluRNA (RNA expression of abundant short, repetitive DNA elements characterized initially by the action of Arthrobacter luteus (Alu) restriction endonuclease) in the RPE of GA patients have been shown to drive NLRP activation and inflammasome expression by MPs and the RPE, respectively (Doyle et al., 2012; Tarallo et al., 2012; Yerramothu et al., 2018). Inflammasome activation is an innate immune system mechanism that can induce pyroptosis, an inflammatory form of programmed cell death usually activated in response to infection, that is distinct to apoptosis and necroptosis (Cookson and Brennan, 2001). NLRP activation of the inflammasome results in cleavage of pro-caspase-1 to caspase-1 and subsequent cleavage of pro-interleukin-1β (pro-IL-1β) and pro-IL-18 into their mature pro-inflammatory forms (Cookson and Brennan, 2001).

In RPE samples from human patients with GA, expression of *Nlrp3* and *Il-18* mRNA was found to be increased relative to normal age-matched controls (Tarallo et al., 2012). Further analysis of protein samples from RPE of GA patients confirmed that in addition to NLRP3 increases, PYCARD (PYD And CARD Domain Containing), pro-caspase-1 and cleaved caspase-1, and downstream effectors of IL-18, including interleukin 1 receptor associated kinase 3 and 4 (IRAK3- and -4) were all increased, suggesting inflammasome activation in the RPE contributes to RPE dysfunction in GA (Tarallo et al., 2012). Another study, which did not isolate RPE specifically, but microdissected regions of photoreceptor and RPE cells from lesion areas of both GA and nAMD and healthy control samples, also found increases in mRNA expression of NLRP3, pro-IL-1β and pro-IL-18 in late-stage AMD (Wang et al., 2016). Studies using RPE cell culture models without MP co-culture suggest that NLRP3 inflammasome activation drives RPE cell damage, including mitochondrial damage and may contribute to AMD pathogenesis (Wang et al., 2016). Although the most likely source of inflammatory mediators, the role of inflammasome activation in MPs in human patients with AMD has not been studied as heavily. A study of the effect of drusen components on human peripheral blood mononuclear cells (PBMCs), which would be the primary source of peripheral MPs with access to the basal RPE, suggests that these cells are a potential source of inflammasome mediators independently of the RPE (Doyle et al., 2012). In this study, drusen components were isolated and used to treat peripheral blood MPs and this induced inflammasome activation and production of IL-1β and IL-18 (Doyle et al., 2012). In line with this, recent single cell RNA-seq analysis of MPs from AMD patients suggested NLRP3 is upregulated in infiltrating inflammatory macrophages (Voigt et al., 2022), implicating these cells as a likely source of inflammasome driven inflammatory mediators.

Further cell culture studies point to an important role of activated MPs in driving inflammation and RPE cell dysfunction and death. Activated MPs have been found to negatively impact RPE function causing a reduction in expression of *Otx2* (orthodenticle homeobox 2), *Rdh5* (retinol dehydrogenase 5), *Ttr* (transthyretin), and *Trf* (transferrin) in cultured RPE cells (Mathis et al., 2017). *Rdh5* and *Ttr* are involved in recycling of vitamin A for rods photoreceptors, while *Trf* is important for iron transport in RPE cells. This decrease in the expression of genes essential for RPE function suggests pro-inflammatory, activated MP interaction with the RPE can contribute to RPE dysfunction (Mathis et al., 2017). Additionally, cell-cell contact between RPE cells and activated MPs can cause apoptosis of RPE cells. Both bone marrow derived macrophages and microglia from mouse retina, that have been activated by inflammatory cytokines such as TNF-α, IFN-γ, and IL-1β, cause cultured mouse RPE cells to enter apoptosis (Devarajan et al., 2016). Similar findings have been reported in co-culture of human RPE cells and human activated monocytes, with a large number of RPE cells entering apoptosis through caspase-3 activation (Elner et al., 1992; Yang et al., 2011). Overall, pro-inflammatory, activated MPs when in contact with RPE cells can cause the death of the RPE, which may lead to dysfunction and failure in nearby remaining RPE cells perpetuating AMD disease progression.

### Angiogenesis and subretinal fibrosis

Mononuclear phagocytes such as microglia have been suggested to play an integral role in angiogenesis during development (Checchin et al., 2006) and they are implicated in new vessel growth in nAMD (Jackson et al., 1997; Ambati and Fowler, 2012; Marneros, 2021). A recent study using single cell RNA-seq on human samples of retina/RPE/choroid from the macula of healthy control and AMD patients (intermediate AMD or CNV, not GA) has shed some light on changes in the MP populations within the eye in AMD and hints at a potential role for MPs in angiogenesis (Voigt et al., 2022). Based on previously described transcriptomes of MPs, three populations of MPs were identified in the retina/RPE/choroid complex: tissue resident macrophages, likely microglia; dendritic cells and inflammatory monocytes (Voigt et al., 2022). Of these, choroidal dendritic cells of AMD patients showed upregulation of both *VEGFA* and matrix metallopeptidase 9 (*MMP9*) (Voigt et al., 2022). Both genes are important for angiogenesis, suggesting that MPs may contribute directly to new vessel growth. The inflammatory macrophage subclass was found to upregulate paired immunoglobulin like 2 receptor alpha (*PILRA*) and ATP binding cassette subfamily A member 1 (*ABCA1*), which like ApoE, is important for cholesterol trafficking. Finally, the vascular cell classes (arteries, veins, and choriocapillaries) showed upregulation of *COL8A1* (collagen type VIII alpha 1 chain) collagen expression, which suggests extracellular matrix remodeling, and upregulation in CFH in the alternative complement pathways, which could modulate MP interaction with the vascular bed (Voigt et al., 2022). The change in interaction between MPs and the vascular bed coupled with an upregulation of angiogenic factors in MPs, such as MMP9 and VEGFA, suggests that MPs may play a prominent role in CNV development, even prior to overt CNV pathology.

Additionally, MPs namely monocytes and derived macrophages recruited in CNV have been suggested to play a role in angiogenesis by secreting pro-angiogenic TNF-α, and IL-1β, which may promote expression of VEGF from the RPE and MPs (Leibovich et al., 1987; Oh et al., 1999; Cousins et al., 2004). The vitreous of patients with nAMD has increased pro-IL-1β and IL-1β relative to age-matched controls (Zhao et al., 2015). While the role of IL-1β in promoting angiogenesis is relatively well supported (Voronov et al., 2014), the role of IL-18 in nAMD has proven slightly controversial. IL-18 may play a protective role in limiting nAMD lesions in mouse models of CNV (Doyle et al., 2012), while other studies have shown it contributes to RPE degeneration (Tarallo et al., 2012). Generally, studies investigating CNV models in mice highlight a role for NLRP3 activation and MP generated IL-1β, but not necessarily IL-18, in nAMD lesion size and development (Marneros, 2021). The vitreous of patients with nAMD has also been found to contain higher levels of growth-related oncogene (GRO), CCL22, and CCL3 (Agrawal et al., 2019). Both CCL22 and CCL3 are chemokines that have been shown to be secreted by macrophages and induce angiogenesis (Agrawal et al., 2019), while GRO is a chemotactic chemokine that has been found to modulate inflammation and angiogenesis in diabetic retinopathy (Schoenberger et al., 2012).

Mononuclear phagocytes have also been implicated in fibrosis, the end stage of nAMD. Fibrosis is characterized as a wound healing process which involves deposition of extracellular matrix proteins driven by activated inflammatory cells and fibroblasts recruited to the damaged site (Little et al., 2018). This process has been suggested to be a primary reason for the failure of anti-VEGF therapies in arresting vision loss in nAMD progression. MPs, especially choroidal macrophages, are associated with CNV and fibrotic lesions in nAMD (Grossniklaus et al., 2000; Cherepanoff et al., 2010; Lad et al., 2015). MPs can trigger the expression of integrins α1 and α5 on the RPE surface via TNF-α production, promoting the RPE cells to migrate on fibronectin and collagen type I leading to fibrotic scarring (Jin et al., 2000; Kent and Sheridan, 2003; Ishikawa et al., 2016). Additionally, MPs have been suggested to directly contribute to fibrosis by transitioning to myofibroblasts (Little et al., 2018). Histological assessment of fibrosis within nAMD lesions and mouse studies of laser induced nAMD suggest that C3 and transforming growth factor beta (TGF-β) can induce MPs to transition to myofibroblasts, with 20–30% of infiltrating macrophages expressing the myofibroblast marker, α-smooth muscle actin, in lesion sites (Little et al., 2018). Treatment of a laser induced mouse model of nAMD with an antifibrotic agent, a novel cinnamyl anthranilate (OCX063), has been shown to reduce lesion size and fibrosis driven via the TGF-β pathway alluding to the potential for treatments targeting TGF-β pathways as an adjunct therapy for nAMD (Brandli et al., 2022).

## Conclusion

Age-related macular degeneration is a multigenic disease associated with a range of environmental risk factors including aging and lifestyle factors. RPE dysfunction and pro-inflammatory MPs have been found to play an important role in the progression of AMD, from early/intermediate stage to advanced forms GA and nAMD. Crosstalk between MPs and the RPE contributes to the pathological changes observed in AMD and the ensuing inflammatory microenvironment can promote RPE cell dysfunction and death, as well as breakdown of blood retinal barrier. The factors that drive MPs to exert a detrimental role in AMD, rather than serving as a protective response against AMD pathology, are many and are still being defined. In the future, determining the MP factors that promote homeostasis and subtle para-inflammation that aid an aging RPE vs. those that promote an overt pro-inflammatory response will likely provide a pathway for developing therapies to slow vision loss in AMD.

## Author contributions

JW, JM, AB, UG, AJ, EF, and KV: manuscript writing and editing. All authors contributed to the article and approved the submitted version.
